# UCHL1, besides leptin and fibronectin, also could be a sensitive marker of the relapsing–remitting type of multiple sclerosis

**DOI:** 10.1038/s41598-023-30237-3

**Published:** 2023-02-28

**Authors:** Ewelina Górska, Marzena Tylicka, Adam Hermanowicz, Ewa Matuszczak, Anna Sankiewicz, Ewa Gorodkiewicz, Justyna Hermanowicz, Elżbieta Karpińska, Katarzyna Socha, Jan Kochanowicz, Marta Jakoniuk, Joanna Kamińska, Evgenija Homšak, Olga Martyna Koper-Lenkiewicz

**Affiliations:** 1 Neurological Private Practice, Stołeczna 7/109, 15-879 Białystok, Poland; 2 Department of Biophysics, Medical University of Białystok, Mickiewicza 2a, 15-089 Białystok, Poland; 3 Department of Pediatric Surgery, Medical University of Białystok, Waszyngtona 17, 15-274 Białystok, Poland; 4 Bioanalysis Laboratory, Faculty of Chemistry, University of Białystok, Ciolkowskiego 1K, 15-245 Białystok, Poland; 5 Department of Clinical Pharmacy, Medical University of Białystok, Mickiewicza 2C, 15-222 Białystok, Poland; 6 Department of Bromatology, Medical University of Białystok, Mickiewicza 2 d, 15-222 Białystok, Poland; 7 Department of Neurology, Medical University of Białystok, M. Skłodowskiej - Curie 24A, 15-276 Białystok, Poland; 8 Department of Invasive Neurology, Medical University of Białystok, M. Skłodowskiej-Curie 24a, 15-276 Białystok, Poland; 9 Department of Clinical Laboratory Diagnostics, Medical University of Białystok, Waszyngtona 15A, 15-269 Białystok, Poland; 10 Department for Laboratory Diagnostics, University Clinical Centre Maribor SI, Ljubljanska Ulica 5, 2000 Maribor, Slovenia; 11 Department for Clinical Biochemistry, Medical Faculty, University of Maribor, Maribor, Slovenia

**Keywords:** Biomarkers, Immunology, Inflammation

## Abstract

Research on the markers of immunoregulatory response in multiple sclerosis (MS) is still of great importance. The aim of our study was the evaluation of leptin, fibronectin, and UCHL1 concentrations as potential biomarkers of a relapsing–remitting type of MS (RRMS). Surface Plasmon Resonance Imaging (SPRI) biosensors were used for the evaluation of proteins concentrations in 100 RRMS patients and 46 healthy volunteers. Plasma leptin, fibronectin, and UCHL1 concentrations were significantly higher in RRMS patients compared to the control group (p < 0.001, respectively). UCHL1 concentration evaluation revealed the highest diagnostic sensitivity (100%) and negative predictive value (100%) in differentiating MS patients from healthy individuals. There was no significant difference in the UCHL1 concentrations depending on the patient’s sex, the presence of relapse within the last 24 months, and the EDSS value (p > 0.05, respectively). In RRMS patients UCHL1 concentration positively correlated with fibronectin levels (r = 0.3928; p < 0.001). In the current cohort of patients plasma UCHL1 concentration was independent of the time of MS relapse and the severity of neurological symptoms. Thus current study may indicate that plasma UCHL1, besides leptin and fibronectin, also could be a promising high-sensitive potential biomarker of relapsing–remitting type of MS. However, these results should be validated with a larger group of patients, taking into account neuroimaging and cerebrospinal fluid analysis data, and by comparing them to patients with other neurological diseases as a control group.

## Introduction

Multiple sclerosis (MS) is a complex autoimmune disease of the central nervous system (CNS) in which an unrelenting attack of the immune system results in extensive demyelination, loss of oligodendrocytes, and axonal degeneration^1,2^. MS is driven by a complex interaction between genetic, immunological, and environmental factors, and mainly affects young adults at the age of 20–40^2,3^. MS patients can develop any neurological signs and symptoms among which the most common are numbness, ataxia, walking difficulties, muscle spasms, bladder or visual problems, fatigue, pain, depression, and dementia^4^.

Studies have indicated that the cytokine-like hormone leptin mostly produced by adipose tissue, may play an important role in MS pathogenesis via the regulation of inflammatory responses^5^. Leptin activates the proliferation of monocytes, enhances the phagocytic activity of macrophages, production of proinflammatory cytokines, and stimulates the proliferation of T cells^6,7^. T cells lead to the recruitment of other inflammatory cells which in turn drive the production of the antibodies and proinflammatory cytokines that destroy the myelin sheath^8^. The interaction between leptin and proinflammatory factors could play a significant role in the pathology of MS^9^. An imbalance in leptin levels can impair immunity and exacerbate autoimmune diseases such as MS and related problems^10,11^.

Autoimmune-mediated inflammation may play a principal role in demyelination^1^. The failure of remyelination in the course of MS has likely been mediated also due to the expression of the extracellular matrix molecule fibronectin which affects remyelination by oligodendrocytes progenitors. Fibronectin levels increase both by leakage from the blood circulation and by production from CNS resident cells^12^.

Research on Ubiquitin C-terminal hydrolase L1 (UCHL1) in the course of MS has not been extensively conducted so far. UCHL1 is a proteolytically stable small protein of neuronal origin whose levels are increased in serum and cerebrospinal fluid (CSF) following traumatic brain injury and correlated with both severities of injury and long-term outcome^12^. This neuron-specific deubiquitinating enzyme is one of the most abundant proteins in the brain and some studies indicated the fundamental importance of UCHL1 in the proper functioning of the nervous system^13^. UCHL1 is involved not only in the process of repairing injured axons and neurons but also takes part in immune reactions^14^.

Controlling the inflammatory reactions may avoid uncontrolled tissue damage, restore peripheral tolerance, and promote remyelination^8^, thus research on the markers of immunoregulatory response in MS is still of great importance. Therefore the aim of our study was the evaluation of leptin, fibronectin, and UCHL1 concentrations as potential biomarkers of a relapsing–remitting type of MS (RRMS). In the next step, we analyzed the relationship of proteins tested with each other and with the age of the patients, the number of years from the first MS symptoms, the number of years from MS diagnosis, the number of relapses within the 24-month observational period, the EDSS value, the EDSS visual functions, the EDSS brainstem functions, the EDSS pyramidal functions, the EDSS cerebellar functions, the EDSS sensory functions, the EDSS bowel/bladder functions, Cerebral functions, and the Ambulation score.

## Material and methods

### Patients

The study population comprised 100 patients who were within a 24-months observational period at the Outpatient Neurological Clinic NZOZ Kendron in Bialystok. Patients eligible for the study met the 2017 McDonald’s criteria for a diagnosis of MS. The MS group was composed of 36 males and 64 females, with a median age of 42 years, and a range of 19–58 years. The inclusion criterion was the relapsing–remitting type of MS (RRMS). This clinical form of MS is characterized by acute attacks (relapses) followed by partial or full recovery (remissions)^5^. During the 2-year follow-up, 0–6 relapses of the disease were observed in MS patients.

All RRMS patients, from whom blood samples were taken in remission have had no worsening of neurological functions between attacks. Exclusion criteria were preexisting infections, diseases that required long-term medication, and other inflammatory or neurological disorders. Clinical and demographical information regarding RRMS patients is summarized in Table 1. The control group was composed of 46 healthy, aged-, sex- and body mass index (BMI)-matched volunteers (15 males, 31 females, with median age of 41 years, range of 19–64 years), subject to the same exclusion criteria.

**Table 1:** Demographical and clinical data of MS patients.

	MS patients (No. = 100)
Sex: Number (%)	Males: 36 (38%)
	Females: 64 (62%)
Age (years)*	42 (32–50)
MS type: Number (%)	RRMS: 100 (100%)
Relapse/Remission: Number (%)	Patients
	In relapse: 0 (0%)
	In remission: 100 (100%)
EDSS*	3.5 (2–4)
The number of years from the first SM symptoms until the blood sample is collected*	8 (3–12)
The number of years from the diagnosis of SM until the blood sample is collected*	4 (1–8)

*MS* multiple sclerosis, *RRMS* relapsing–remitting multiple sclerosis, *EDSS* Kurtzke’s Expanded Disability Status Scale.

*Data is presented as Median with 25th and 75th percentiles.

Approval for this study was obtained from the Local Bioethics Committee (Permission No: APK.002.282.2021). Procedures were under the ethical standards set by the Declaration of Helsinki given by the World Medical Association. Written informed consent was obtained from all study participants. According to Polish medical standards, the collection of CSF through a lumbosacral puncture can only be performed in a hospital setting, as it is an invasive procedure. Therefore, the Local Bioethics Committee gave permission only to collect patients’ blood. It was not possible to collect CSF from MS patients and healthy volunteers.

### Leptin, fibronectin, and UCHL1 concentrations evaluation

#### Reagents

Recombinant human UCHL1 protein and rabbit monoclonal antibody (R&D Systems, USA), recombinant human leptin protein and rabbit anti-leptin antibody (Abcam, United Kingdom), fibronectin from human plasma and rabbit anti-fibronectin antibody (Sigma-Aldrich, Germany) were used.

The reagents described in the previous study^16^ were utilized in the current study. „The cysteamine hydrochloride, N-ethyl-N′-(3-dimethylaminopropyl) carbodiimide (EDC), N-hydroxysuccimide (NHS) were from Sigma-Aldrich, Germany, absolute ethanol, acetic acid, hydrochloric acid, sodium hydroxide, sodium chloride, sodium carbonate, sodium acetate were from POCh, (Poland) HBS-ES buffer pH = 7.4 (0.01 M HEPES, 0.15 M sodium chloride, 0.005% Tween 20, 3 mM EDTA), Phosphate Buffered Saline (PBS) pH = 7.4, carbonate buffer pH = 8.5 from BIOMED (Poland) were used as received. Aqueous solutions were prepared with Milli-Q water (Simplicity® Millipore). Argon N 5.0 with a content Ar ≥ 99,999% was used (AIR LIQUIDE Polska Sp.z o.o., Poland).”

#### Procedure for determination of UCHL1, fibronectin, and leptin concentrations with SPRI biosensors

Surface Plasmon Resonance Imaging (SPRI) biosensors were used for the evaluation of UCHL1, fibronectin, and leptin concentrations in plasma samples. The procedure for the preparation of the biosensors for determinations and the research methodology are presented in the previous articles, the same as the schematic diagram of the SPRI apparatus^15–18^.

A rabbit monoclonal antibody specific for human UCHL1, a rabbit polyclonal antibody for fibronectin, and a rabbit monoclonal anti-leptin antibody as a bio-recognition element of biosensors were used^15–18^.

For the biosensor preparation, the gold chip was modified with layer cysteamine. Next, the antibody solution in a PBS buffer (concentration value: 10 ug/mL for UCHL1, 4 µg/mL for fibronectin, and 60 ng/ml for leptin) was activated with NHS (50 mM) and EDC (200 mM) in a carbonate buffer (pH = 8.5) environment and was placed on the layer of cysteamine. The chip prepared in this way was incubated at 37 °C for 1 h. After incubation, the biosensor was rinsed with water and HBS-ES buffer solution (pH = 7.4). Plasma samples were diluted with PBS buffer directly before measurement (for determination of UCHL1: 2–10 times, fibronectin: 1000 times, and leptin: 2–5 times). The diluted samples of plasma were spotted directly on the active place of the prepared biosensor and incubated at room temperature for 10 min. After this time, unbound molecules were removed from the biosensor surface by washing with water and HBS-ES buffer^15–18^.

The SPRI signal was measured from the images of active sites obtained by the CCD camera before and after analyte-receptor interaction. The NIH ImageJ 1.32 software with actualization to version 1.50i (Wayne Rasband National Institutes of Health, USA, http://imagej.nih.gov/ij, Java 1.8.0_181 (32-bit), 4990 K of 640 MB (< 1%)) was used to process the images ^15,17^. Based on the obtained SPRI signals, the concentrations were read from the previously prepared calibration curves. The linear range of curves was 0.5–3.0 ng/mL (r^2^ = 0.9867) for UCHL1, 5.00–400 ng/mL (r^2^ = 0.9968) for fibronectin, and 0.1–10 ng/mL for leptin (r^2^ = 0.9954). Control of non-specific binding on the surface of each biosensor was performed by using sites of the biosensor with PBS buffer^15–18^.

### Statistical analysis

Statistical analysis was conducted using the STATISTICA PL release 13.3 Program (StatSoft Inc., Tulsa, USA) and GraphPad Prism 8.4.3 software (GraphPad Software, San Diego, USA). All the results are presented as median with 25th and 75th percentiles. Shapiro–Wilk’s test of normality was used for data distribution analysis. The non-Gaussian data were analyzed using the non-parametric Mann–Whitney U test or the Kruskal–Wallis H test. Spearman’s Rank method was used for the correlation coefficient analysis. The differences were deemed statistically significant when p < 0.05.

### Ethics approval and consent to participate

Approval for this study was obtained from the Bioethics Committee of the Medical University of Białystok (No: APK.002.282.2021). Procedures were following the ethical standards set out in the Declaration of Helsinki put out by World Medical Association. We received consent from individual patients who participated in the present study.

## Results

Leptin, fibronectin, and UCHL1 concentrations were detectable in all plasma samples obtained from MS as well as the control subjects.

### Plasma leptin concentration results

**Figure 1:**
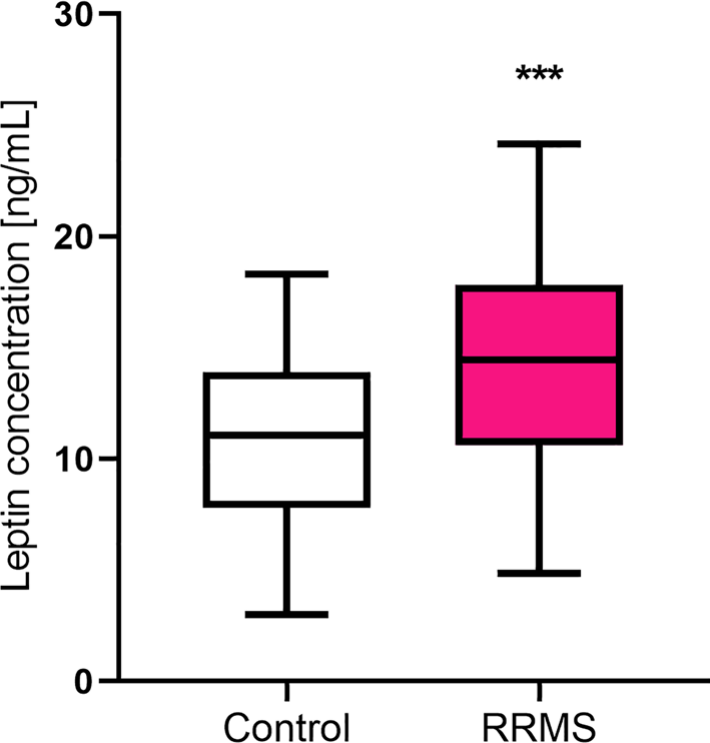
Plasma Leptin concentration in relapse-remitting multiple sclerosis patients (RRMS) as compared to the healthy control individuals. Statistical significance ***p ≤ 0.001.

Plasma leptin concentration was significantly higher in MS patients (1.30-fold increase) as compared to the control group (Fig. 1, Table 2). However, there was no difference in the concentration of this marker depending on the patient’s sex (p > 0.05). Leptin concentration did not differ between patients who had a relapse within the last 24 months compared to those who did not and depending on the EDSS value (p > 0.05) (Tables 3, 4). Also, we did not find a correlation between leptin concentration and fibronectin and UCHL1 concentration as well as the age of the patients, the number of years from the first MS symptoms, the number of years from MS diagnosis, the number of relapses within the 24-month observational period, the EDSS value, the EDSS visual functions, the EDSS brainstem functions, the EDSS pyramidal functions, the EDSS cerebellar functions, the EDSS sensory functions, the EDSS bowel/bladder functions, Cerebral functions, and the Ambulation score (p > 0.05).

**Table 2:** Plasma leptin, fibronectin, and UCHL1 concentrations in MS patients as compared to the control group (C).

	Leptin [ng/mL]		Fibronectin [ng/mL]		UCHL1 [ng/mL]	
	MS	C	MS	C	MS	C
Median	**14.44**	**11.08**	**232.29**	**126.93**	**16.89**	**4.07**
Percentiles (25–75%)	10.63–17.82	7.97–13.88	213.57–252.40	111.29–144.10	13.66–19.06	3.57–4.34
p-value*	**p < 0.001**		**p < 0.001**		**p < 0.001**	
Fold increase	**1.30**		**1.83**		**4.15**	

Significant values are in bold.

*MS* multiple sclerosis.

*A p-value < 0.05 is considered to show a significant difference between groups (according to the Mann–Whitney U test).

**Table 3:** Plasma leptin, fibronectin, and UCHL1 concentration depending on the presence of relapse within the last 24 months in MS patients.

	The last relapse was more than 24 months ago	The last relapse was within 24 months	p-value
Leptin [ng/mL]	13.14 (10.39–17.49)	13.85 (10.65–16.88)	p = 0.8836
Fibronectin [ng/mL]	231.70 (213.25–246.80)	234.43 (214.04–251.76)	p = 0.5579
UCHL1 [ng/mL]	16.40 (13.60–18.66)	16.82 (13.60–19.01)	p = 0.6090

*MS* multiple sclerosis. Data is presented as Median with the Percentiles (25-75%).

**Table 4:** Plasma leptin, fibronectin, and UCHL1 concentration depending on the EDSS value in MS patients.

	From no disability with only minimal signs to minimal disability	From moderate to relatively severe disability	From disability affecting full daily activities to the need for assistance with walking and working	p-value
	EDSS: 1–2.5	EDSS: 3–4.5	EDSS: 5–6.5	
Leptin [ng/mL]	14.22 (10.33–17.67)	12.60 (10.39–15.84)	13.78 (10.65–17.48)	p = 0.6450
Fibronectin [ng/mL]	232.36 (211.03–246.08)	230.36 (217.36–251.76)	226.86 (214.36–244.79)	p = 0.8931
UCHL1 [ng/mL]	16.14 (13.33–18.95)	15.67 (13.54–18.27)	17.03 (16.18–18.18)	p = 0.3496

*MS* multiple sclerosis, *EDSS* Kurtzke’s Expanded Disability Status Scale. Data is presented as Median with the Percentiles (25-75%).

In the control group, plasma leptin concentration did not differ between males and females (p > 0.05). Also, we did not find a correlation between leptin concentration and fibronectin and UCHL1 concentration as well as the age of the control individuals (p > 0.05, respectively).

### Plasma fibronectin concentration results

**Figure 2:**
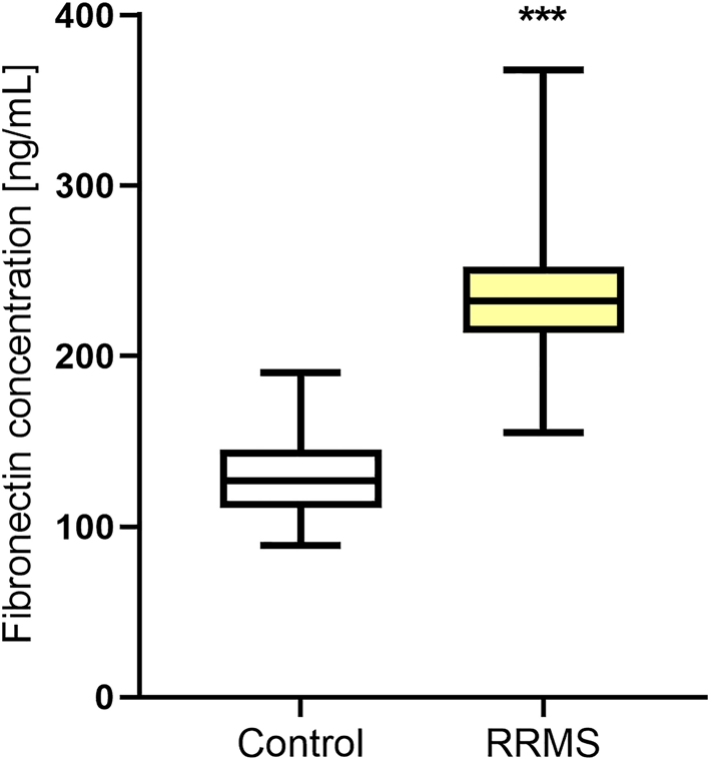
Plasma Fibronectin concentration in relapse-remitting multiple sclerosis patients (RRMS) as compared to the healthy control individuals. Statistical significance ***p ≤ 0.001.

Plasma fibronectin concentration was significantly higher in MS patients (1.83-fold increase) as compared to the control group (Fig. 2, Table 2). We did not observe differences between the concentration of this parameter in the samples taken from female and male patients (p > 0.05). Fibronectin concentration did not differ between patients who had a relapse within the last 24 months compared to those who did not and depending on the EDSS value (p > 0.05) (Tables 3, 4). We also did not find a correlation between fibronectin concentration and leptin concentration as well as the age of the patients, the number of years from the first MS symptoms, the number of years from MS diagnosis, the number of relapses within the 24-month observational period, the EDSS value, the EDSS visual functions, the EDSS brainstem functions, the EDSS pyramidal functions, the EDSS cerebellar functions, the EDSS sensory functions, the EDSS bowel/bladder functions, Cerebral functions, and the Ambulation score (p > 0.05).

Plasma fibronectin concentration in the control group did not show a sex difference (p > 0.05). No correlation was also found between fibronectin concentration and UCHL1 concentration as well as the age of control individuals (p > 0.05, respectively).

### Plasma UCHL1 concentration results

**Figure 3:**
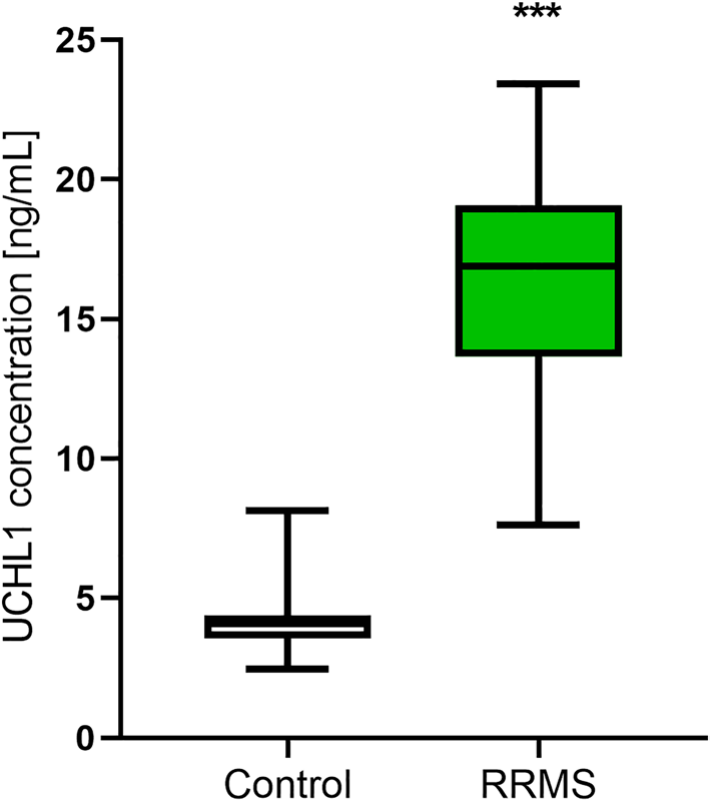
Plasma UCHL1 concentration in relapse-remitting multiple sclerosis patients (RRMS) as compared to the healthy control individuals. Statistical significance ***p ≤ 0.001.

**Figure 4:**
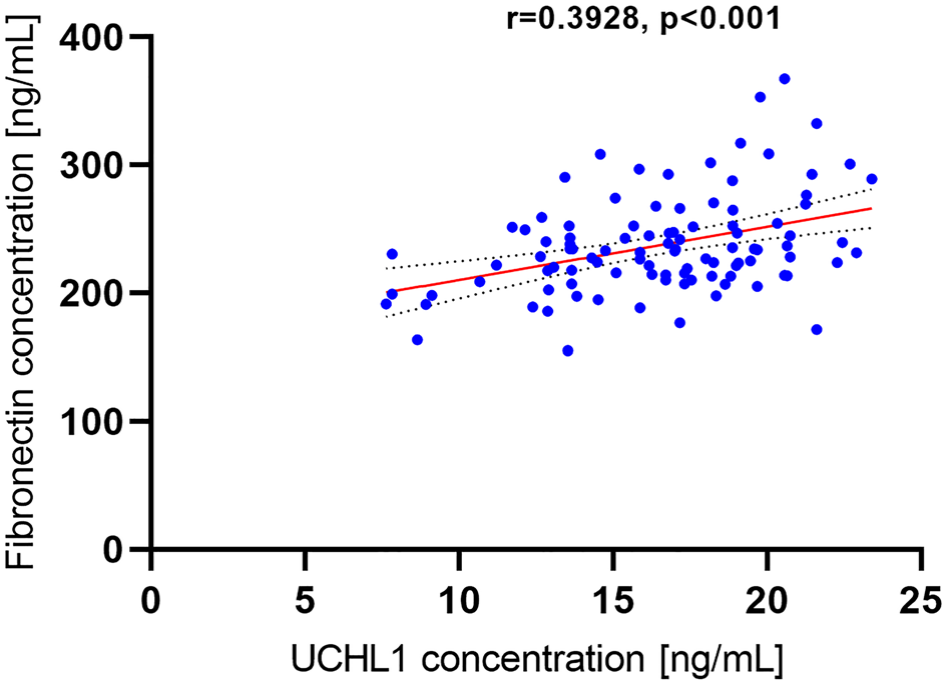
The correlation coefficient between fibronectin and UCHL1 concentration in relapse-remitting multiple sclerosis patients.

We noticed that plasma UCHL1 concentration was significantly higher (a 4.15-fold increase) in patients with MS than in the control group (Fig. 3, Table 2). We did not observe differences between the concentration of this marker in females and males with MS (p > 0.05). UCHL1 concentration did not differ between patients who had a relapse within the last 24 months compared to those who did not and depending on the EDSS value (p > 0.05) (Tables 3, 4). We also did not find a correlation between UCHL1 concentration and the age of the patients, the number of years from the first MS symptoms, the number of years from MS diagnosis, the number of relapses within the 24-month observational period, the EDSS value, the EDSS visual functions, the EDSS brainstem functions, the EDSS pyramidal functions, the EDSS cerebellar functions, the EDSS sensory functions, the EDSS bowel/bladder functions, Cerebral functions, and the Ambulation score (p > 0.05). However, closer inspection showed a positive, average correlation between UCHL1 and fibronectin concentration in MS patients (r = 0.3928; p < 0.001) (Fig. 4).

In the control group, no difference between plasma UCHL1 concentration in the samples taken from female and male patients was found (p > 0.05). Moreover, no correlation between UCHL1 and the age of control individuals was detected (p > 0.05).

### Plasma UCHL1 is the most sensitive marker of the relapsing–remitting type of multiple sclerosis

**Figure 5:**
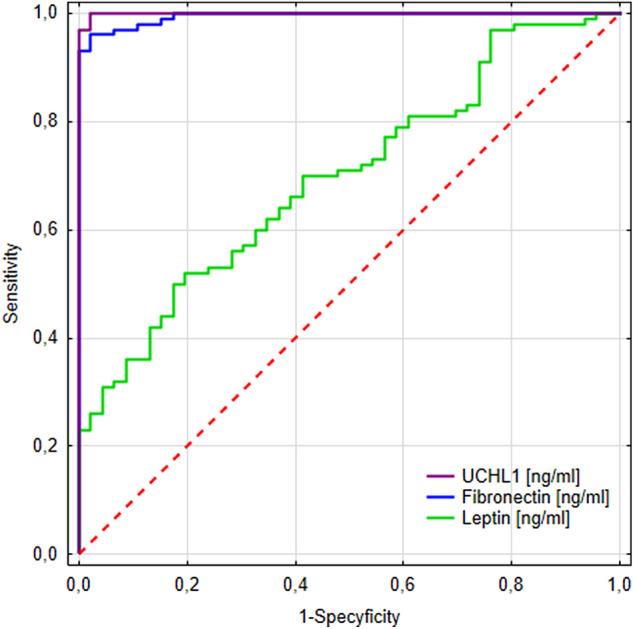
Areas under the ROC curves (AUCs) for plasma leptin, fibronectin, and UCHL1 evaluation in differentiating relapse-remitting multiple sclerosis patients from healthy individuals. For plasma leptin the AUC = 0.698, cut-off = 14.49 ng/ml; for plasma fibronectin the AUC = 0.994, cut-off = 0.94 ng/ml; for plasma UCHL1 the AUC = 0.999, cut-off = 7.63 ng/ml.

Plasma UCHL1 was the most useful in differentiating MS patients from healthy individuals. It revealed the highest AUC (0.999), diagnostic sensitivity (100%) as well as negative predictive value (100%). Also, the diagnostic specificity (98%), positive predictive value (99%), and diagnostic accuracy (93%) were very high. The AUCs for leptin and fibronectin were also statistically higher than AUC = 0.5, which indicates their diagnostic usefulness for differentiating MS patients from healthy individuals (Table 5, Fig. 5).

**Table 5:** Diagnostic usefulness of plasma leptin, fibronectin, and UCHL1 evaluation in differentiating MS patients from healthy individuals.

	Cut-off	Youden index	AUC ± SE	Se [%]	Sp [%]	PPV [%]	NPV [%]	ACC [%]	p-value*
Leptin [ng/mL]	14.49	0.33	0.698 ± 0.044	50	83	86	43	60	p < 0.0001
Fibronectin [ng/mL]	0.94	185.76	0.994 ± 0.003	96	98	99	92	97	p < 0.0001
UCHL1 [ng/mL]	7.63	0.98	0.999 ± 0.001	100	98	99	100	93	p < 0.0001

*ACC* diagnostic accuracy, *AUC* area under the ROC curve, Cut-off (based on the highest Youden Index), *MS* multiple sclerosis, *NPV* negative predictive value, *PPV* positive predictive value, *SE* standard error, *Se* diagnostic sensitivity, *Sp* diagnostic specificity.

*A p-value < 0.05 is considered to be a statistically significant.

## Discussion

Multiple Sclerosis (MS) is a chronic autoimmune disease^10^. It is indicated that various genetic, environmental, and lifestyle factors contribute to the disease’s onset and progression^9^. In most MS patients the disease manifests itself in a relapsing–remitting form (RRMS). This type of MS is typically associated with a clinical event and subsequently complete or partial recovery^19^.

There are some pieces of evidence that human leptin, which the main role is the regulation of adipose tissue mass and energy balance, may participate also in MS pathogenesis^20^. Leptin production can be induced by inflammatory mediators such as TNF-α and IL-1. Moreover, leptin together with C-reactive protein, IL-6, and IL-1 can act as an early acute-phase reactant^21^. The current study presented that plasma leptin levels were significantly higher in MS patients as compared to the healthy control subjects, which confirms that it could be recognized as a pro-inflammatory cytokine affecting immune response in MS and indicate this protein as a potential circulating marker useful in disease diagnosis. Previously also Bahrami et al.^20^ noticed a significant difference in leptin levels between healthy volunteers and the MS group. Moreover, Düzel et al.^22^ in their study on adipokines in multiple sclerosis showed that the levels of leptin were significantly higher in relapsing–remitting MS patients compared with the healthy control group. Frisullo et al.^5^, who examined the effect of disease activity on leptin concentration in relapsing–remitting MS, observed higher leptin levels in RRMS patients in remission than in controls. Additionally, they found that leptin concentration is higher in remission than in relapse of MS. Although several studies suggested a statistically significant difference in leptin levels between female and male MS patients^5,7,20^, our observation did not confirm these results, which is in agreement with the research of Fahmi et al. and Eftekhari et al.^6,23^. We also did not observe any significant difference between leptin concentration in females and males from the control group.

Inflammation in the course of MS leads also to the loss of myelin (demyelination) and incomplete remyelination which are pathological characteristics of the disease. The failure of remyelination is likely mediated by changes in the extracellular signaling environment. Stoffels et al.^12^ presented that extracellular matrix (ECM) molecule fibronectin aggregates are observed in MS lesions and are responsible for remyelination impairment. Fibronectin, a high-molecular-weight multifunctional glycoprotein, regulates cell adhesion, spreading, migration, proliferation, as well as apoptosis^24,25^. It exists as soluble plasma disulfide-linked dimeric form produced by hepatic stellate cells (HSCs) and as a cellular-derived insoluble component of ECM produced by a wide range of cells such as fibroblasts, myocytes, chondrocytes, and synovial cells^24–26^. Fibronectin is expressed at low levels in healthy adult tissues and increases in response to injury or inflammatory processes, which are presented within the CNS in the course of MS^27^. According to Stoffels et al., the best way to positively modulate the remyelination process in MS is to avoid fibronectin aggregation and promote fibronectin clearance^12^. Previous studies by van Horssen et al., Sobel et al., and Satoh et al.^28–30^ also demonstrated the expression of fibronectin in chronically demyelinated MS lesions. Our results have indicated that also circulating levels, not only fibronectin aggregates, may indicate the process of inflammation in the course of MS. We observed that plasma fibronectin concentration increased 1.83-fold in patients with RRMS in comparison to the control group. Interestingly, we also found a positive correlation between fibronectin and UCHL1 concentrations. However, in the control group, we did not present such a correlation.

The potential role of UCHL1 in MS has not been studied so far, therefore in the current study, we examined this protein as a potential candidate biomarker for MS diagnosis. UCHL1 is mostly expressed in neuroendocrine cells and CNS and under physiological conditions constitutes about 2% of soluble proteins in the brain^14^. It is hypothesized that the measurement of circulating neuroglial proteins levels such as UCHL1 in the CSF or blood can be used to detect and assess injury to the nervous system in neurological diseases and trauma, such as MS, amyotrophic lateral sclerosis (ALS), stroke, or traumatic brain injury (TBI)^31,32^. According to Sjölin et al.^32^ analysis of UCHL1 concentration in the blood may reflect the size and location of CNS damage. Especially, that Li et al.^31^ have indicated that in ALS patients CSF UCHL1 concentrations significantly correlate with its serum levels (r = 0.7709, p < 0.0001). However, proper interpretation of such data could be possible only by having detailed knowledge about the distribution of UCHL1 in the CNS^32^. Thus the Authors create a map of the UCHL1 concentration in 17 different anatomical regions of the CNS in humans. In their study post-mortem tissue homogenates were obtained from deceased donors without a history of neurological disease. The authors presented, that throughout the cerebrum, the concentration of UCHL1 was higher in the cortex than in white matter. The highest concentration of UCHL1 was found in the hippocampus and the lowest in the cerebellum^32^.

In the current cohort of RRMS patients plasma UCHL1 concentration increased the most (4.15-fold) in comparison to the healthy control group. This could be justified by the fact, that UCHL1 has biological significance in many processes including inflammation and neuronal injury^14^. This enzyme plays an important role in the removal of excessive, oxidized, and misfolded proteins during neuropathological conditions. Brophy et al.^33^, demonstrated that UCHL1 levels are increased following traumatic brain injury (TBI) and the enzyme growth is correlated with both severities of injury and long-term outcome. Papa et al.^34^ showed that UCHL1 as a novel biomarker has the potential to determine injury severity in TBI. However, in contrast to the above data, our previous study on the markers of brain injury caused by mild head trauma (MHD) presented that UCHL1 is not a useful marker of MHD due to less tissue damage in comparison to TBI^35^. Despite this, it should be mentioned that our earlier research on UCHL1 as a potential marker showed a higher concentration of this deubiquitinating enzyme in the case of cryptorchidism, thermal injury, and acute appendicitis^36–38^.

This study is the first, that showed the possibility of UCHL1 being a promising high-sensitive potential biomarker of RRMS, especially since the sensitivity of plasma UCHL1 concentration evaluation in differentiating MS patients from healthy individuals was very high (100%). We have demonstrated that there is no statistically significant difference in the concentrations of plasma UCHL1 depending on the patient’s sex, the presence of relapse within the last 24 months, and the EDSS value. Plasma UCHL1 concentration also did not correlate with the age of the patients, the number of years from the first MS symptoms, the number of years from MS diagnosis, the EDSS visual functions, the EDSS brainstem functions, the EDSS pyramidal functions, the EDSS cerebellar functions, the EDSS sensory functions, the EDSS bowel/bladder functions, Cerebral functions, and the Ambulation score. These data may indicate that plasma UCHL1 concentration is independent of the time of MS relapse and independent of the severity of neurological symptoms. In the control group UCHL1 concentration was independent of the sex and age of healthy individuals. However, these findings should be further validated on a larger cohort of patients and regarding in relationship to neuroimaging and cerebrospinal fluid analysis data. Here we also have reported for the first time, that the increasing concentration of fibronectin, which has an essential role in the pathological immune response contributing to MS^27^, correlated with the concentration of UCHL1 in the course of RRMS.

One limitation of the study is that the evaluation of biomarkers has not been tested in patients with other neurological diseases as a control group. Therefore, to definitively determine if the candidate markers are specific to MS, additional research should be conducted in neurological conditions other than MS.

## Conclusion

Our current study showed, that besides leptin and fibronectin, also plasma UCHL1 could be a promising high-sensitive potential biomarker of relapsing–remitting MS, as it was the most useful in differentiating RRMS patients from healthy individuals. Plasma UCHL1 concentration evaluation revealed the highest AUC (0.999), diagnostic sensitivity (100%) as well as negative predictive value (100%) in differentiating MS patients from healthy individuals. Moreover, in the current cohort of patients plasma UCHL1 concentration was independent of the time of MS relapse and the severity of neurological symptoms. Thus it could be potentially recognized as an independent circulating marker of RRMS. However, these results should be validated with a larger group of patients, taking into account neuroimaging and cerebrospinal fluid analysis data, and by comparing them to patients with other neurological diseases as a control group.

## Data Availability

The datasets generated and analyzed during the current study are not publicly available but all are kept at the Medical University of Bialystok and are available from the corresponding author on reasonable request.

## Abbreviations

BMI: Body mass index
CSF: Cerebrospinal fluid
CNS: Central nervous system
EDSS: Kurtzke’s Expanded Disability Status Scale
HSCs: Hepatic stellate cells
IL: Interleukin
MHD: Mild head trauma
MS: Multiple sclerosis
RRMS: A relapsing–remitting type of MS
TBI: Traumatic brain injury
TNF-α: Tumor necrosis factor alfa
UCHL1: Ubiquitin C-terminal hydrolase L1

## References

[1] McQualter JL, Bernard CCA (2007). Multiple sclerosis: A battle between destruction and repair. J. Neurochem..

[2] Misrielal C, Mauthe M, Reggiori F, Eggen BJL (2020). Autophagy in multiple sclerosis: Two sides of the same coin. Front. Cell. Neurosci..

[3] Haase S, Linker RA (2021). Inflammation in multiple sclerosis. Ther. Adv. Neurol. Disord..

[4] Pegoretti V (2020). Inflammation and oxidative stress in multiple sclerosis: Consequences for therapy development. Oxid. Med. Cell. Longev..

[5] Frisullo G (2007). The effect of disease activity on leptin, leptin receptor and suppressor of cytokine signalling-3 expression in relapsing-remitting multiple sclerosis. J. Neuroimmunol..

[6] Fahmi RM, Kamel AE, Elsayed DA, Zidan AA, Sarhan NT (2021). Serum levels of leptin and adiponectin in patients with multiple sclerosis. Egypt. J. Neurol. Psychiatry Neurosurg..

[7] Evangelopoulos ME, Koutsis G, Markianos M (2014). Serum leptin levels in treatment-naive patients with clinically isolated syndrome or relapsing-remitting multiple sclerosis. Autoimmune Dis..

[8] Calahorra L, Camacho-Toledano C, Serrano-Regal MP, Ortega MC, Clemente D (2022). Regulatory cells in multiple sclerosis: From blood to brain. Biomedicines.

[9] Rijnsburger M, Djuric N, Mulder IA, de Vries HE (2021). Adipokines as immune cell modulators in multiple sclerosis. Int. J. Mol. Sci..

[10] Moharami S (2022). Investigation of serum levels of orexin-A, transforming growth factor β, and leptin in patients with multiple sclerosis. J. Clin. Lab. Anal..

[11] Cojocaru M, Cojocaru IM, Siloşi I, Rogoz S (2013). Role of Leptin in Autoimmune Diseases. MAEDICA.

[12] Stoffels JMJ (2013). Fibronectin aggregation in multiple sclerosis lesions impairs remyelination. Brain.

[13] Bilguvar K (2013). Recessive loss of function of the neuronal ubiquitin hydrolase UCHL1 leads to early-onset progressive neurodegeneration. Proc. Natl. Acad. Sci. U. S. A..

[14] Matuszczak E, Tylicka M, Komarowska MD, Debek W, Hermanowicz A (2020). Ubiquitin carboxy-terminal hydrolase L1—Physiology and pathology. Cell Biochem. Funct..

[15] Sankiewicz A (2015). Development of surface plasmon resonance imaging biosensors for detection of ubiquitin carboxyl-terminal hydrolase L1. Anal. Biochem..

[16] Sankiewicz A, Romanowicz L, Pyc M, Hermanowicz A, Gorodkiewicz E (2018). SPR imaging biosensor for the quantitation of fibronectin concentration in blood samples. J. Pharm. Biomed. Anal..

[17] Sankiewicz A, Hermanowicz A, Grycz A, Łukaszewski Z, Gorodkiewicz E (2021). An SPR imaging immunosensor for leptin determination in blood plasma. Anal. Methods.

[18] Gorodkiewicz E, Sankiewicz A, Laudański P (2014). Surface plasmon resonance imaging biosensors for aromatase based on a potent inhibitor and a specific antibody: Sensor development and application for biological material. Cent. Eur. J. Chem..

[19] Huitema MJD, Schenk GJ (2018). Insights into the mechanisms that may clarify obesity as a risk factor for multiple sclerosis. Curr. Neurol. Neurosci. Rep..

[20] Bahrami E (2014). Leptin hormone level in serum of opticospinal, neuromyelitisoptica and multiple sclerosis patients. Clin. Exp. Neuroimmunol..

[21] Matarese G, Procaccini C, De Rosa V (2008). The intricate interface between immune and metabolic regulation: A role for leptin in the pathogenesis of multiple sclerosis?. J. Leukoc. Biol..

[22] Düzel B, Tamam Y, Çoban A, Tüzün E (2019). Adipokines in multiple sclerosis patients with and without optic neuritis as the first clinical presentation. Immunol. Invest..

[23] Eftekhari E, Etemadifar M, Ebrahimi A, Baradaran S (2013). The relation between peptide hormones and sex hormone in patients with multiple sclerosis. Iran. J. Neurol..

[24] Patten J, Wang K (2021). Fibronectin in development and wound healing. Adv. Drug Deliv. Rev..

[25] Liu XY (2016). Fibronectin expression is critical for liver fibrogenesis in vivo and in vitro. Mol. Med. Rep..

[26] Dalton CJ, Lemmon CA (2021). Fibronectin: Molecular structure, fibrillar structure and mechanochemical signaling. Cells.

[27] Stoffels JMJ, Zhao C, Baron W (2013). Fibronectin in tissue regeneration: Timely disassembly of the scaffold is necessary to complete the build. Cell. Mol. Life Sci..

[28] van Horssen J, Bö L, Dijkstra CD, de Vries HE (2006). Extensive extracellular matrix depositions in active multiple sclerosis lesions. Neurobiol. Dis..

[29] Sobel RA, Mitchell ME (1989). Fibronectin in multiple sclerosis lesions. Am. J. Pathol..

[30] Satoh JI, Tabunoki H, Yamamura T (2009). Molecular network of the comprehensive multiple sclerosis brain-lesion proteome. Mult. Scler..

[31] Li R, Wang J, Xie W, Liu J, Wang C (2020). UCHL1 from serum and CSF is a candidate biomarker for amyotrophic lateral sclerosis. Ann. Clin. Transl. Neurol..

[32] Sjölin K (2022). Distribution of five clinically important neuroglial proteins in the human brain. Mol. Brain.

[33] Brophy GM (2011). Biokinetic analysis of ubiquitin C-terminal hydrolase-L1 (UCH-L1) in severe traumatic brain injury patient biofluids. J. Neurotrauma.

[34] Papa L (2010). UCH-L1 is a novel biomarker for severe traumatic brain injury in human. Crit. Care Med..

[35] Tylicka M (2020). BDNF and IL-8, but not UCHL-1 and IL-11, are markers of brain injury in children caused by mild head trauma. Brain Sci..

[36] Toliczenko-Bernatowicz D (2018). Overexpression of ubiquitin carboxyl-terminal hydrolase 1 (UCHL1) in boys with cryptorchidism. PLoS ONE.

[37] Matuszczak E (2017). Overexpression of ubiquitin carboxyl-terminal hydrolase L1 (UCHL1) in serum of children after thermal injury. Adv. Med. Sci..

[38] Matuszczak E (2018). Concentration of UHCL1 in the serum of children with acute appendicitis, before and after surgery, and its correlation with CRP and prealbumin. J. Investig. Surg..

